# Epigenetic liquid biopsies for minimal residual disease, what’s around the corner?

**DOI:** 10.3389/fonc.2023.1103797

**Published:** 2023-04-04

**Authors:** Andrew D. Johnston, Jason P. Ross, Chenkai Ma, Kim Y. C. Fung, Warwick J. Locke

**Affiliations:** Human Health, Health and Biosecurity, CSIRO, Westmead, NSW, Australia

**Keywords:** MRD, epigenetics, liquid biopsy, ccfDNA (circulating cell-freeDNA), cancer, ctDNA (circulating tumor DNA), DNA methylation, fragmentomics

## Abstract

Liquid biopsy assays for minimal residual disease (MRD) are used to monitor and inform oncological treatment and predict the risk of relapse in cancer patients. To-date, most MRD assay development has focused on targeting somatic mutations. However, epigenetic changes are more frequent and universal than genetic alterations in cancer and circulating tumor DNA (ctDNA) retains much of these changes. Here, we review the epigenetic signals that can be used to detect MRD, including DNA methylation alterations and fragmentation patterns that differentiate ctDNA from noncancerous circulating cell-free DNA (ccfDNA). We then summarize the current state of MRD monitoring; highlight the advantages of epigenetics over genetics-based approaches; and discuss the emerging paradigm of assaying both genetic and epigenetic targets to monitor treatment response, detect disease recurrence, and inform adjuvant therapy.

## 1 Introduction

Minimal residual disease (MRD) refers to a small number of cancer cells remaining in a patient’s body after oncological treatment with curative intent. MRD does not cause clinical symptoms and is not detectable by traditional methods such as imaging (e.g., CT, MRI, PET, ultrasound, X-ray, etc.) or abnormal blood serum protein levels. MRD assays must be sensitive enough to detect as little as 1 cancerous cell in a background of 1 million noncancerous cells. This extreme sensitivity makes MRD assay development complex, but overcoming this barrier imbues MRD assays with the potential for much earlier detection of cancer recurrence than traditional methods, and thus earlier intervention with adjuvant chemotherapy or second and third-line treatments.

**Figure 1:**
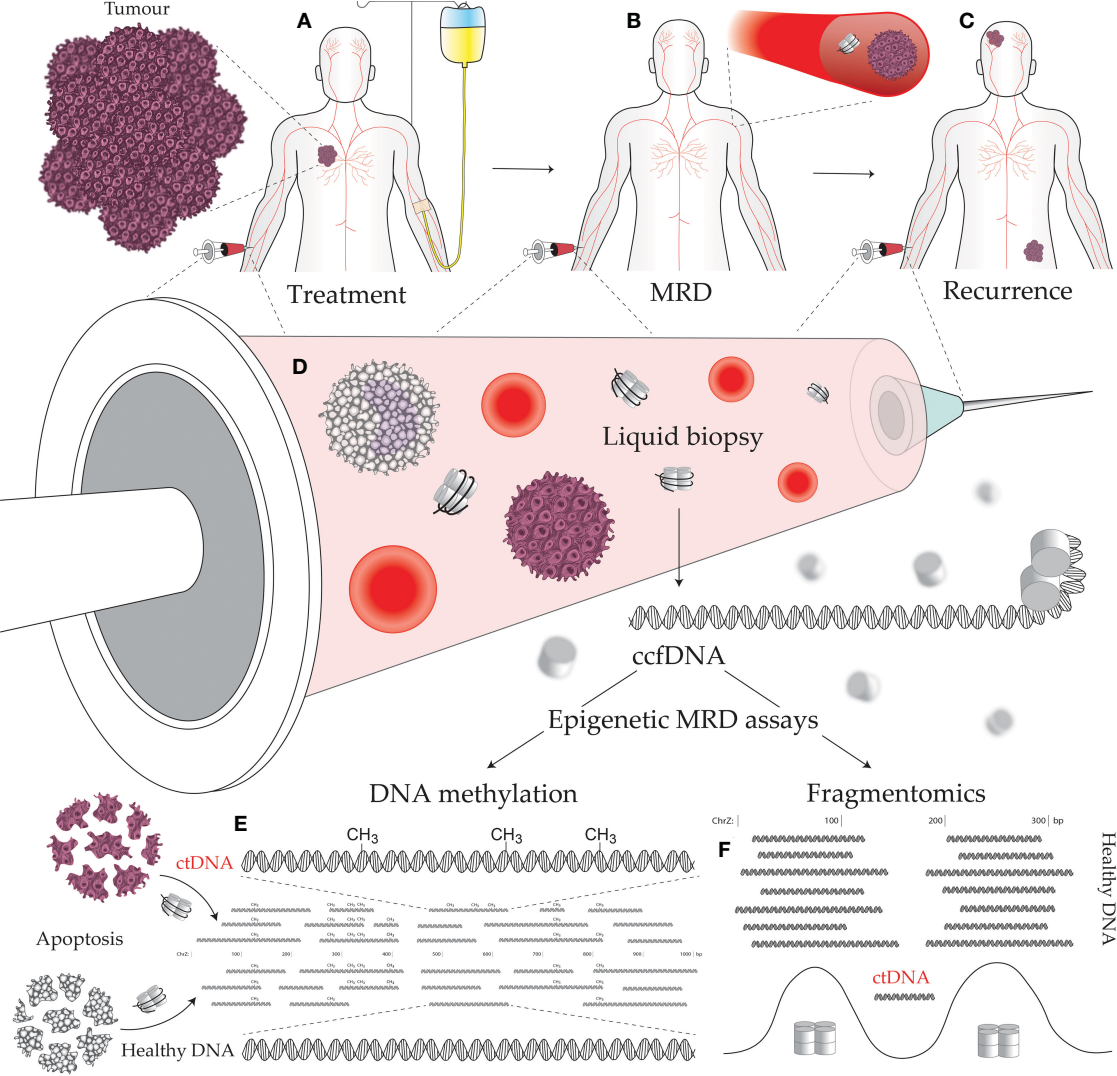
Minimal residual disease (MRD) epigenetic assays and their use across a cancer patient journey. **(A)** Establishing the molecular targets for MRD assays can be performed prior to treatment initiation. A decline in the level of the assayed biomarker suggests efficacious treatment and reduced tumor burden. **(B)** The continued presence of a tumor biomarker after curative intent treatment, accompanied by no visible signs of a tumor, indicates the presence of MRD. **(C)** Tracking with an MRD assay over time is useful for early detection of cancer recurrence, as the level of the assayed biomarker will increase as a tumor remerges. **(D)** Liquid biopsies can be performed on a variety of bodily fluids including blood, urine, saliva, lymph, and cerebrospinal fluid. Circulating tumor DNA (ctDNA) contains epigenetic alterations that can separate it from the cancer’s normal tissue counterpart and circulating cell-free DNA (ccfDNA) derived from noncancerous sources. Cancer-specific epigenetic alterations include: **(E)** aberrant DNA methylation, and **(F)** altered fragmentomic signals (i.e., changes in ctDNA fragment lengths, end sequences, and nucleosome-associated genomic positioning).

Detection of MRD indicates the failure of treatment to eliminate all cancerous cells which implies greater probability of future disease recurrence. Monitoring MRD signals during the remission period allows early detection of disease progression (1). Assaying for MRD, therefore, offers a means to monitor, predict, and detect cancer recurrence, as well as stratify patients for adjuvant therapy and predict their response to treatment (**Figures 1A-C**) (2, 3). However, with the lack of a solid tumor to sample, traditional biopsy methods cannot be applied to MRD assays. Instead, MRD detection must make use of circulating tumor-derived entities such as circulating tumor cells (CTCs), extracellular vesicles, and circulating tumor DNA (ctDNA). These circulating molecules and cells shed by tumors are a rich source of information on cancer biology, featuring much of the genetic and epigenetic abnormalities that drive tumorigenesis and treatment response (**Figure 1D**) (4, 5). Such assays can be used to match patients to effective treatments by detecting clinically actionable variations (e.g., drug resistance) within the detected cancer cells and their molecular products, thus avoiding invasive procedures and the morbidities of ineffectual therapies. These novel diagnostic assays have utility in predicting both MRD and cancer recurrence. A positive MRD test after surgery can inform the decision for: (i) further surgery with curative intent; (ii) adjuvant chemotherapy; and/or (iii) increased surveillance, including imaging such as an FGD-PET scan. Whereas positive recurrence indicated by a quantitatively increasing signal over time can inform the decision for: (i) increased surveillance; and (ii) a switch to secondary/tertiary therapy based on the clonal evolution of the tumor burden, as determined by the assayed biomarkers being tracked (i.e., somatic mutations).

## 2 Epigenetics in oncology

### 2.1 DNA methylation

DNA methylation is a covalent modification of the DNA strand that, in mammals, occurs almost exclusively within the sequence context of cytosine followed by guanosine (CpG) dinucleotides. The genomic acquisition of DNA methylation is an essential developmental process with diverse roles including repressive associations at gene promoters and mobile genetic elements or, conversely, increased transcriptional activity when found within gene bodies [reviewed in Greenberg & Bourc’his (6)]. Genome wide deregulation of DNA methylation is a universal feature of cancer development, typified by global loss of methylation (particularly in repetitive sequences) (7) and localized foci of hypermethylation (8). While genetic alterations have proved useful in liquid biopsy and cancer management (including early cancer detection, prognosis, monitoring therapeutic response and detecting MRD), epigenetic alterations occur more frequently in cancer development and are typically more universal across tumors than specific somatic mutations (9–13). Aberrant DNA methylation is an early event in carcinogenesis that can both drive and be driven by tumor biology/treatments, is associated with a diverse range of biological impact, and can be targeted by liquid biopsies to detect specific cancers (**Figure 1E**) (14). Moreover, DNA methylation profiling in cancer has demonstrated an epigenetic role in hormone receptor signaling and response to endocrine therapy (15), prognosis (16, 17), metastasis (18), response to chemotherapy (19), and key aspects of tumor biology (20).

Most DNA methylation-based liquid biopsies target cancer-specific aberrations that separate cancer from its normal tissue counterpart. However, within healthy tissues DNA methylation exhibits highly cell type specific patterns across the genome (21). This untapped layer of epigenomic information provides a unique signature that can be targeted without the need for detectable cancer-specific aberrations. Despite the significant alteration of the epigenome during carcinogenesis, the underlying tissue-specific methylation patterns linked to cellular identity are still detectable (22). Applications of this approach have focused on the identification of tumor type, particularly in cases of cancer of unknown primary (23). However, the presence of circulating cell-free DNA (ccfDNA) originating from a source other than haemopoietic cells, blood vessel endothelial cells, or liver hepatocytes is exceeding rare in healthy individuals and thus indicative of pathology on its own (21, 24). Therefore, tissue-specific biomarkers could potentially be used in MRD assays to detect signals that span molecular subtypes of tumors that share a tissue of origin. Importantly, these targets are also unlikely to diminish due to tumor adaptation in response to treatment. To this point, recent academic research has begun to focus on exploiting tissue specificity along with ccfDNA originating outside the cancer. The process of cancer growth is highly disruptive to the organ/tissue in which it occurs. This results in elevated levels of cell death in the surrounding “healthy” tissues due to increased stresses causing inflammation and/or deformation of normal tissue structures (25–27). Further development of these methods is required before clinical application extends beyond the research domain; however, such novel approaches show significant promise in the management of advanced/recurrent disease.

### 2.2 Fragmentomics

The term “fragmentomics” describes the study of ccfDNA fragmentation patterns and the use of these patterns to decern biologically relevant information such as nucleosome positioning. Although few assays to-date utilize ccfDNA fragmentation patterns, numerous properties of ctDNA compared to healthy ccfDNA instill fragmentomics with the potential for use in oncological assays (**Figure 1F**). These properties include: (i) region-specific differences in ctDNA fragments lengths (28); (ii) shorter ctDNA fragment length distributions (29); (iii) fragment end sequence alterations in ctDNA (30); and (iv) altered nucleosome positioning in cancer (31). The most notable applications of fragmentomics so far reside around ctDNA fragment lengths. Mouliere et al. (29) demonstrated that selecting shorter ccfDNA fragments (between 90–150) greatly enriches for ctDNA and improves detection of clinically actionable mutations and copy number alterations. This enrichment technique should be a key consideration in MRD design going forward, given the extreme sensitivity required by assays to detect MRD. Beyond enrichment, Cristiano et al. (28) accurately discriminated 215 healthy individuals from 208 cancer patients (AUC = 0.94) using a machine learning model trained on ccfDNA fragment size and coverage across the genome, thus demonstrating the potential for region-specific ctDNA fragment lengths to be used as cancer biomarkers. Delfi Diagnostics is currently pursuing commercialization of this technology with a focus on lung cancer and is in the process of recruiting 15,000 individuals for their CASCADE-LUNG clinical validation study.

As for nucleosome positioning, ccfDNA fragmentation patterns downstream of transcription start sites have been shown to reflect differential nucleosome positioning between expressed and unexpressed genes (32). These ccfDNA read depth coverage patterns match with gene expression signatures of hematopoietic cells (33). Similarly, ccfDNA-derived nucleosome spacing footprints around DNase I hypersensitive sites (e.g., occupied transcription factor binding sites) match most strongly with hematopoietic cell lines (31). These results are consistent with ccfDNA methylation analyses, which show that ccfDNA from healthy donors originates from white blood cells (55%), erythrocyte progenitors (30%), vascular endothelial cells (10%) and liver hepatocytes (1%) (21). In cancer, high levels of ctDNA can create a nucleosome footprint that diverges from these healthy sources and instead corresponds to a cancer’s tissue of origin. For example, ccfDNA-derived nucleosome spacing has been used to infer the tissues of origin of late-stage tumors (31) and ccfDNA coverage patterns have been used to accurately classify expression levels of genes with somatic copy number gains in metastatic cancer patients (33). These findings demonstrate the potential for ctDNA fragmentation signatures to be used as cancer biomarkers. Moreover, nucleosome positioning is cell-type specific (34, 35), and nucleosome linker DNA lengths are up to 7 base pairs shorter in transcriptionally active chromatin versus inactive genes/compartments (31, 36). We envisage regions of the genome that are active in noncancerous ccfDNA contributing tissues and inactive in a tumor’s tissue of origin, and vice versa. Such regions should possess a subset of nucleosome positions that are out-of-phase between these two sources due to differences in nucleosome spacing, thus producing a subpopulation of ctDNA fragments positioned at the breakpoints of “healthy” ccfDNA. In which case, future MRD assays could potentially exploit tissue-specific nucleosome positioning to identify an underlying cancer signal at low ctDNA concentrations.

Going forward, the most sensitive and universal MRD assays will likely combine these epigenomic approaches. Until recently, the combination of DNA methylation and fragmentomics in a single assay was not possible due DNA methylation detection relying on bisulfite conversion (37). Bisulfite conversion causes DNA damage that erodes the fragmentomic signals embedded within ccfDNA fragments. However, the recent advent of enzymatic methylation conversion technology allows highly accurate methylation detection to be performed without this damage and the resulting loss of fragmentomic signals (38). Therefore, it is now possible to use next-generation methylation sequencing to detect both DNA methylation and fragmentomic biomarkers. At the very least, this means ctDNA sized-based enrichment can now be used to improve the detection sensitivity of DNA methylation-based MRD assays. However, enzymatic conversion also means differentially methylated regions, region-specific fragment length changes, and nucleosome positioning biomarkers can now all be incorporated into a single hybridization capture-seq panel.

## 3 MRD assays: Products on-market, clinical trials, and emerging technologies

The marketplace for MRD liquid biopsy assays is new, but several MRD diagnostic tests have now received early regulatory approvals (**Table 1**). In 2021, the Natera Signatera test for MRD was granted Breakthrough Device Designation (BDD) status by the FDA for an intended use in Stage I-IV colorectal cancer. This approach uses whole-exome sequencing of primary tumors along with matched normal blood samples to prepare bespoke personalized multiplex PCR assays. In total, 16 patient-specific somatic variants are selected to track using next-generation sequencing (NGS) (39, 40). The Inivata RaDaR test for residual disease and recurrence has received a Conformité Européenne (CE) mark for use in Europe and was granted FDA BDD status in 2021 (41, 42). Again, exome-sequencing informs the design of a secondary somatic mutation PCR panel and tracks up to 48 mutations in blood using NGS. These two-staged approaches are stated by their respective companies to take between 2 and 4 weeks for assay design and up to 1 week turnaround on subsequent blood draws. Finally, the FoundationOne Tracker test was awarded BDD status in 2022 and combines Foundation Medicine’s patient-specific NGS tumor profiling with the multiplex PCR design of Natera (43). This test is used to detect and track MRD, assess a patient’s response to therapy, and monitor for relapse following curative intent therapy.

**Table 1:** Tests for mininmal residual disease currently avaliable or in development.

Test/Company	Technology/method	Clinical Applications	Development Stage/Regulatory Approval	Citations
Signatera Company: Natera	Somatic mutation detection in ctDNA using multiplex-PCR NGS assays Personalised targets from whole-exome sequencing of primary tumors	MRD and recurrence in: - Colorectal cancer (Stage I-IV) - Epithelial ovarian cancer (Stage I-IV)	BDD status (USA)	(39, 40)
RaDaR Company: Inivata	Somatic mutation detection in ctDNA using multiplex-PCR NGS assays Personalised targets from whole-exome sequencing of primary tumors	MRD and recurrence in: - Breast cancer	CE mark (Europe) BDD status (USA)	(41, 42)
FoundationOne Tracker Company: Foundation Medicine	Somatic mutation detection in ctDNA using bespoke multiplex-PCR NGS assays Personalised targets from whole-exome sequencing of primary tumors	MRD, treatment response and recurrence in: - Colorectal cancer - Bladder cancer	BDD status (USA)	(43)
Sentinel Trail Company: Strata Oncology	Somatic mutation detection in ctDNA using bespoke multiplex-PCR NGS assays Personalised targets from whole-exome sequencing of primary tumors	Treatment response and recurrence in: - Stage 1-3 solid tumors	Clinical Trial (NCT05082701)	(44)
brPROPHET Company: Burning Rock Biotech	Somatic mutation detection in ctDNA using bespoke hybridzation capture poanels and NGS. Personalised targets from whole-exome sequencing of primary tumors	MRD and recurrence in: - Colorectal cancer	Ungoing clinical studies	(45)
PhasED-seq Company: Foresight Diagnostics	Detection of phased varients (PVs), where two or more mutations on the same sequenced fragment of ctDNA PV panel assembled from whole-genome sequences of 2,538 tumors	MRD and recurrence in: - B-cell lymphomas	Developing as LDT	(46)
CORRECT-MRD II Company: Exact Sciences	Highly sensitive variaent detection using targeted linear pre-amplification of ccfDNA, UMI ligation, then somatic mutation detection using bespoke multiplex-PCR NGS assays. Personalised targets from whole-genome sequencing of primary tumors.	MRD and recurrence in: - Colorectal cancer (Stage II & III)	Clinical Trial (NCT05210283)	(47)
ECLIPSE Company: Guardant Health	Somatic mutation, DNA methylation and fragmentomic profiling of ccfDNA. 500 kB hybridization capture panel (LUNAR) for cancer detection using NGS.	Early detection, MRD and recurrence in: - Colorectal cancer	Clinical Trial (NCT04136002)	(48)
ctDNA Methylation Sequencing for Myeloma Company: MethylGene Tech	ctDNA methylation sequencing	MRD and recurrence in: - Multiple myeloma	Clinical Trial (NCT05578625)	(49)
Targeted methylation platform Company: GRAIL	ctDNA targeted methylation detection	MRD and recurrence	In development	(50)
Colvera Company: Clinical Genomics	real-time PCR test for detecing DNA methylation of BCAT1 and IKZF1 genes	MRD and recurrence in: - Colorectal cancer	Currently offered as an LDT	(51, 52)
Bladder EpiCheck Company: Nucleix	Multiplex DNA methylation-based PCR assay	Tumor recurrence in: - Bladder cancer	CE mark (Europe)	(53, 54)
ColonAiQ Company: Singlera Genomics	Six-plex methylation PCR test	MRD, treatment response and recurrence in: - Colorectal cancer	Clinical Trial (NCT05444491) Clinical Trial (NCT05536089)	(55)

While there are only a few somatic mutation-based MRD panels in the marketplace, there are a host currently in development and undergoing clinic trials. For example, Strata Oncology are also exploring a two-staged tumor-informed panel design in their Sentinel trial for cancer recurrence (44). Burning Rock Biotech has developed brPROPHET, a personalized capture panel-based approach for MRD detection and are currently working with BeiGene to progress this assay to clinical studies (45). Foresight Diagnostics (46) claims that Phased Variant Enrichment & Detection Sequencing (PhasED-seq), where two or more mutations occur on the same strand of DNA, offers more sensitivity for MRD. The company intends to develop a CLIA validated assay and market the assay as a lab-development test (LDT). Exact Sciences is running the 750 patient CORRECT-MRD II study to validate their MRD and recurrence test for stage II and III colorectal cancer. This is based on the Target Digital Sequencing (TARDIS) approach that Exact Sciences licensed from The Translational Genomics Research Institute (47).

Notably, genetic mutations are not broadly shared across cancers and the two-stage solutions to this shortcoming are limited by tumor heterogeneity and the volume of a tissue biopsy. That is, because only a subset of a tumor is sequenced there is still a high probability that mutations will be missed. However, a multi-omics approach can augment this shortcoming. The greater frequency and universality of epigenetic changes makes them more sensitive and universal markers for evidence of cancer after curative intent treatment, and somatic mutation testing highlights actionable variants that can inform subsequent treatment. This promise of MRD assays that incorporate epigenetics with somatic mutation testing is being realized. Guardant Health has combined somatic mutations, DNA methylation and fragmentomics into their LUNAR panel, which they have progressed into clinical trials for both primary detection and MRD of early-stage colorectal cancer (48).

Multiple companies are also progressing purely epigenetic-based MRD panel approaches. MethylGene is undertaking a clinical trial on multiple myeloma patients that will utilize DNA methylation sequencing of ctDNA for MRD detection (49). While GRAIL has concentrated on bringing the DNA methylation-based Galleri test to market for early detection of cancer, in 2021 the company also formed a deal with Amgen, AstraZeneca, and Bristol Myers Squibb to evaluate its technology for the detection of MRD and early recurrences (50). This expansion of use case by GRAIL points to the versatility of ultra-sensitive liquid biopsy assays. Fast turnaround and inexpensive serial epigenetic tests can identity MRD and recurrence early. A positive result provides the evidence to undertake imaging and to order large NGS panels to study clonal evolution of the tumor and indicate modes of treatment.

While most product development for MRD is concentrated around sequencing large panels of markers, simple DNA methylation-based PCR tests can also be highly efficacious. Colvera, developed by Clinical Genomics, is available as an LDT in the USA and has Medicare coverage for MRD and recurrence monitoring of colorectal cancer. This methylated ctDNA test detected 66.0% of recurrence, significantly higher than the 31.9% sensitivity of carcinoembryonic antigen (CAE), the current standard of care (51), and also has potential for identifying MRD due to treatment failure (52). Another PCR test is Bladder EpiCheck offered by Nucleix. This multiplex DNA methylation-based PCR assay is for monitoring of tumor recurrence in conjunction with cystoscopy in patients previously diagnosed with bladder cancer (53, 54). Also in development, Singlera Genomics is recruiting colorectal cancer patients to run clinical trials on their ColonAiQ six-plex methylation PCR test for MRD and cancer recurrence (55).

Interestingly, a simple well-designed PCR test can have adequate diagnostic power when compared to a large NGS panel. This is illustrated when comparing the rates of primary detection of colorectal cancer across the GRAIL-sponsored circulating cell-free genome atlas (CCGA) (27) study (Trial identifiers: NCT02889978 and NCT03085888) and Colvera trial data (56) (Trial identifier: ACTRN12611000318987). Both are large prospective observational studies and both report similar rates of detection across the four cancer stages, despite the GRAIL technology sequencing the entire ccfDNA repertoire and the Clinical Genomics test being only a two-gene PCR assay. This suggests the liquid biopsy detection of cancer, including MRD detection, may be primarily limited by the presence of any ctDNA in the peripheral blood, not the features nor breadth of the detection technology.

## 4 Conclusion

Liquid biopsy is a powerful, multifaceted, and minimally invasive method for MRD detection and hence for monitoring therapeutic response, disease recurrence, or patient resistance to therapy. Although a relatively new field, liquid biopsy assays are showing promising results when compared to current standards of care. Two-staged somatic mutation approaches, where tumor sequencing informs MRD assay targets, allow clinicians to track actionable mutations and monitor for signs of treatment resistance. However, the time required to establish theses assays does not fit well with the patient journey, as clinicians need to know sooner than 5-6 weeks whether a patient is not responding to treatment. Therefore, epigenetic-based MRD assays are preferable at treatment onset, as epigenetic changes are typically more widespread and likely to be shared among a greater number of cancers than somatic mutations. With the recent advent of enzymatic methylation conversion, targeted sequencing approaches combining DNA methylation, fragmentomics and machine learning will likely come to predominate. However, DNA methylation-based PCR tests currently offer the most cost-effective solution within this niche. In fact, a compelling paradigm may be the combination of inexpensive serial testing with a DNA methylation-based assay, then following a positive MRD/recurrence result with a broad NGS-based somatic mutation panel to identify actionable mutations.

## Author contributions

AJ, JR, CM, KF, and WL wrote and revised this review. All authors contributed to the article and approved the submitted version.
